# Use of Branded Food Composition Databases for the Exploitation of Food Fortification Practices: A Case Study on Vitamin D in the Slovenian Food Supply

**DOI:** 10.3389/fnut.2021.775163

**Published:** 2022-01-04

**Authors:** Sanja Krušič, Maša Hribar, Edvina Hafner, Katja Žmitek, Igor Pravst

**Affiliations:** ^1^Nutrition Institute, Nutrition and Public Health Research Group, Ljubljana, Slovenia; ^2^Biotechnical Faculty, University of Ljubljana, Ljubljana, Slovenia; ^3^VIST—Faculty of Applied Sciences, Ljubljana, Slovenia

**Keywords:** vitamin D, food fortification, fortification, food supply, Europe, Slovenia

## Abstract

Vitamin D deficiency is a worldwide public health concern, which can be addressed with voluntary or mandatory food fortification. The aim of this study was to determine if branded food composition databases can be used to investigate voluntary fortification practices. A case study was conducted using two nationally representative cross-sectional datasets of branded foods in Slovenia, collected in 2017 and 2020, and yearly sales data. Using food labeling data we investigated prevalence of fortification and average vitamin D content, while nutrient profiling was used to investigate overall nutritional quality of the foods. In both datasets, the highest prevalence of vitamin D fortification was observed in meal replacements (78% in 2017; 100% in 2020) and in margarine, corresponding to high market share. Other food categories commonly fortified with vitamin D are breakfast cereals (5% in 2017; 6% in 2020), yogurts and their imitates (5% in 2017; 4% in 2020), and baby foods (18% in both years). The highest declared average content of vitamin D was observed in margarine and foods for specific dietary use (7–8 μg/100g), followed by breakfast cereals (4 μg/100g), while the average content in other foods was below 2 μg/100g. Only minor differences were observed between 2017 and 2020. Major food-category differences were also observed in comparison of the overall nutritional quality of the fortified foods; higher overall nutritional quality was only observed in fortified margarine. Our study showed that branded food composition databases are extremely useful resources for the investigation and monitoring of fortification practices, particularly if sales data can also be used. In the absence of mandatory or recommended fortification in Slovenia, very few manufacturers decide to add vitamin D, and even when this is the case, such products are commonly niche foods with lower market shares. We observed exceptions in imported foods, which can be subject to fortification policies introduced in other countries.

## Introduction

Vitamin D (VitD) is a fat-soluble vitamin family usually encompassing ergocalciferol (D2) and cholecalciferol (D3) (1, 2). It is a pro-hormone with a well-established role in musculoskeletal health and other functions (3–5). Discussion about the importance of sufficient VitD status during the COVID-19 epidemic should also be mentioned (6–9), but consensus has not yet been reached on this topic.

The chemical structure of VitD was described in the early years of the last century. After small quantities were first found in butterfat and cod liver oil (10), it was later observed that it can be biosynthesized after sun exposure (11). It is now well established that the exposure of skin to sufficiently intensive ultraviolet B (UVB) sunlight is a key source of VitD for humans (12); UVB photons enter the skin and photolyze 7-dehydrocholesterol into previtamin D3, which is then transformed into vitamin D3 (13, 14). However, the cutaneous biosynthesis is affected by various personal and environmental factors (15–19), and when it is insufficient to ensure adequate VitD status, dietary intake of VitD becomes of major importance. Unfortunately, both sources combined are commonly not enough, making VitD deficiency one of the most frequent micronutrient deficiencies globally (20). The situation in Slovenia, a mid-latitude European country (45° and 46°N), is also far from perfect; considerable seasonal variations in VitD status were reported; with about 80% of the adult population having insufficient VitD status, and about 40% VitD deficiency during winter time (21).

The poor VitD status can be improved with increased dietary intake of VitD. We can distinguish three dietary sources of this vitamin: (a) VitD naturally present in foods, such as cod liver oil and oily fish, such as sardines, mackerel, and salmon (22, 23) (however, very few foods are good VitD sources); (b) medicines or dietary supplements that contain VitD; (c) foods that are enriched or fortified with VitD. While some researchers carefully distinguish between food enrichment and fortification, based on the food matrix and/or purpose (24), herein we only use the term “fortification”. According to the World Health Organization (WHO), food fortification is a “practice of deliberately increasing the content of an essential micronutrient (i.e., vitamins) in a food to improve the nutritional quality of the food supply and provide a public health benefit with minimal health risk” (25). In general, VitD content in foods can be increased with different strategies. Most commonly used fortification approach is simply adding VitD to processed foods. On one hand the nutritional quality of food crops can be improved through plant breeding, agronomic practices, or modern biotechnology (26–30). Examples of these approaches are feeding hens with VitD to increase its content in eggs, likewise with livestock animals in relation to meat, and UV exposure of mushrooms or yeast (29, 31). In Europe, UV-treated yeast, which is a good source of vitamin D2, was approved as a novel food in various food categories (32, 33).

In the European Union (EU), the addition of micronutrients to foods was harmonized in 2006 with the adoption of Regulation 1925/2006 on the addition of vitamins, minerals, and other certain substances to foods (34, 35). The legislation enables the adding of micronutrients to foods in cases of deficiency in the population, possibly improving the nutritional status in the population or certain groups of the population, or if their use is scientifically supported. Fortification is not allowed for unprocessed foods (fruit, vegetables, meat, fish, etc.) and alcoholic beverages. The legislation also defines the chemical substances that can be used for fortification. In the case of VitD, both cholecalciferol and ergocalciferol can be used (36).

To efficiently improve VitD intake in the general population with the fortification of foods, several issues should be considered. Fortified foods must contain a sufficient amount of VitD, they should be consumed by the majority of the population, and they must meet specific standards for bioavailability, storage stability, and cooking conditions. They should also not exceed the amount of VitD that could have adverse effects (27, 37). The United States of America (USA) and some other industrialized countries, such as Great Britain, introduced VitD fortification back in the 1930s and 1940s (26). Because early efforts were mainly focused on the prevention of rickets in children, cow's milk was first selected as a fortification matrix. This was followed by other foods, including dairy products, margarine, hot dogs, peanut butter, and others (38). In the Great Britain in the 1960s and in the USA in the 1980s, uncontrolled or accidental high intakes of VitD through fortified products occurred. Poor monitoring and large excesses of fortification of some dairy products led to hypercalcemia in some cases, and as a result, fortification of dairy products with VitD was then banned in some countries (39–41).

Although voluntarily addition of vitamins to foods is harmonized across the EU (36), fortification practices vary between countries. In most countries, including Slovenia, there are no requirements for mandatory fortification and no formal recommendations on this topic. However, some countries adapted their national policies or guidelines. In Finland for example, the voluntary fortification of foods was recommended in 2003, when the government encouraged the addition of VitD to margarine/fat spreads (10 μg/100 g of food) and fluid milk products (0.5 μg/100ml of milk) (35, 42, 43). The recommended fortification content doubled in 2010 (44). Although this was a voluntary option, it was followed by most manufacturers, and a notable reduction in VitD deficiency was observed in the population (42, 45).

Foods fortified with vitamins are commonly labeled with various nutrition and health claims (46), which can be very attractive for consumers (47). Previous research highlighted issues related with the overall nutritional quality of such foods, which can be high in energy, fat, sugar or salt content (48–50), because this area is still not regulated in the EU (51). Similar might apply for the foods fortified with specific vitamins, but this area has not yet been investigated.

Considering the high prevalence of VitD deficiency in Slovenia (52), the government is searching for the most feasible policy approaches to address this public health problem. While the COVID-19 epidemic has resulted in notable changes in dietary behaviors (53), and also increased the supplementation of VitD in the general population (54), a feasible long-term solution is needed to ensure an optimal VitD status in the general population. A possible route forward is the introduction of national VitD fortification guidelines, but data about existing fortification practices is needed before the introduction of an evidence-based policy decision.

With thousands of different foods in the food supply, the investigation of voluntary fortification practices is very challenging. Branded food composition databases have been shown to be excellent resource for investigating nutritional quality and the content of certain nutrients and additives in the food supply (55–57). The objective of this case study was, therefore, to explore the possibility of the use of the nationally representative branded food composition database to investigate VitD supplementation practices. Our goals were to identify food categories with added VitD and to determine the prevalence of fortification and the typical amount of added VitD in those categories. Nutrient profiling was used to investigate the overall nutritional quality (healthiness) of the fortified foods, in comparison with non-fortified foods. Study utilized two cross-sectional datasets (from the years 2017 and 2020), compiled within the national research program “Nutrition and Public Health” and the EC-funded “Food Nutrition Security Cloud” project (FNS-Cloud; www.fns-cloud.eu). Market-share differences were addressed with the use of nation-wide 12-month sales data, provided by major food retailers. Fortification practices were investigated using both datasets, while nutrient profiling was conducted on the 2017 dataset.

## Materials and Methods

### Data Collection and Categorization

The source of branded food composition data was the Slovenian Composition and Labeling Information System (CLAS). CLAS is an online tool for monitoring the supply of the prepacked foods in Slovenia, maintained by the Nutrition Institute (Ljubljana, Slovenia) (58). The tool was developed within the national research program “Nutrition and Public Health”, funded by a Slovenian research agency. In Slovenia, this tool was first tested for a food supply study in 2015 on specific food categories (55, 59), while the first complete food supply study (on all categories of prepacked foods) was conducted in year 2017 (56, 60), and repeated in year 2020. Within this monitoring program, food labels of prepacked foods in the Slovenian food supply are photographed. In the CLAS tool, the data about the nutritional composition and food ingredients are extracted. To ensure representativeness, the data was collected from shops of all the major retailers with a nationwide market. In 2017, five retailers were included (Mercator, Spar, Tuš, Hofer, and Lidl), while in 2020, we additionally included the retailer Eurospin. The data collections were conducted in Ljubljana (Slovenia).

For this case study, we utilized food composition datasets compiled in years 2017 and 2020. EAN (EAN; European Article Number) barcode numbers were used as unique food identifiers. This approach enabled us to avoid duplicate entries of foods, which are available in shops of different retailers. The data collection approach was previously described in detail (56, 60). In short, the collected information included the product's name, the list of the ingredients, nutritional values, packaging volume, price, and barcode number. Each product was assigned to one of the 16 parent categories and 57 categories using a classification system developed within the Global Food Monitoring Initiative (GFMI) (61). The food categorization system was nationally adapted with additional sub-categories on the third level (57).

While the CLAS datasets include all the prepacked foods available in the selected grocery stores at the time of sampling, this case study only included food categories in which we found products fortified with VitD. Altogether, the CLAS contained 21,090 products in 2017, and 28,028 products in year 2020. VitD fortification was identified with the food ingredients lists as provided by the manufacturers on the food labels. All items in the dataset in which the ingredient list wording contained relevant VitD-related terms (“vitamin D,” “cholecalciferol,” “calciferol,” “ergocalciferol,” “D2,” or “D3”) were manually checked by a researcher to identify products fortified with VitD. After exclusion of food supplements, the following food categories included products with added VitD in either the 2017 or 2020 dataset: beverages; bread and bakery products; cereal and cereal products; confectionery; dairy and imitates; edible oils and oil emulsions; foods for special dietary use; sauces and spreads. All further analyses were done on these food categories. Number of foods in the datasets containing these food categories were 13,393 in 2017, and 16,064 in year 2020. The content of VitD was taken from the nutrition declaration on the label and corresponded to the composition of food as sold (without further preparation). We did not conduct laboratory analyses of foods.

Reformulation practices were investigated on a sub-sample of products, which were found in both the 2017 and 2020 datasets. Matching was conducted using barcode numbers. If the 2020 dataset contained a similar product with a different barcode number, it was not included into comparison. In the original datasets with all food categories, *N* = 10,034 foods (47.6% of the 2017 sample; 35.8% of the 2020 sample) were found in both years. In the dataset with only the above-mentioned food categories with VitD added to the foods, *N* = 6,534 foods were found in both years (48.8% of the 2017 sample and 40.7% of the 2020 sample).

Previously described sale-weighting approach (57) was used to account for different market-shares of different foods. This approach was applied to all items, for which 12-month national sales data was available. The 12-month volume sales data were provided by the major retailers, covering a majority of the national food retail market. These sales data were connected with the datasets using barcode numbers. In the above-mentioned selected food categories, the sales data were available for 9,258 (69.1%) products in 2017, and for 10,923 (68.0%) in 2020 dataset.

Evaluation of the overall nutritional quality of foods was investigated using two nutrient profiling models, namely the WHO Europe (WHOE) profile (62), and the Nutri-Score (NS) (63). WHOE was developed to restrict the advertising of unhealthy foods to children, while NS is a scheme for front-of-package nutrition labeling, which assigns foods into five grades A-B-C-D-E; dark green grade A is assigned to products with highest overall nutrition quality, and dark orange grade E to those with the lowest quality. This scheme is implemented for voluntarily use in some European countries (France, Germany, Belgium, Luxembourg, Netherlands, Switzerland and Spain) (64). We compared nutrient profiles of VitD fortified foods with those that were not fortified. Nutrient profiling methodology is presented in details elsewhere (65). In short, nutrient profile was determined with consideration of the nutritional composition of food, standardized to 100 g. In addition to the content of energy, total sugars, saturated fats, salt, dietary fiber and proteins, the percentage of fruits/vegetables/pulses/nuts/specific oils was also considered. Information provided on food labels were used for calculations (65, 66). Foods passing WHOE criteria (permitted for marketing) were assigned as having higher nutritional quality. Considering that both used nutrient profiling models are category specific, comparisons were done within (sub)categories. Analyses was done using 2017 dataset; out of the 13,393 products in the original dataset, *N* = 2,327 foods were excluded: 828 due to missing food composition information that is needed for profiling, and 1,499 for other reasons, i.e., food category not covered in the nutrient profiling algorithms for example foods for specific dietary use, which are excluded form algorithms because of specific nutritional needs of children) (67).

### Data Processing and Statistical Analyses

The food composition data were processed using Microsoft Analysis Services Client Tools 13.0, Microsoft SQL Server Management Studio 13.0, Microsoft Excel 2019 (Microsoft, Redmond, Washington, DC, USA), Microsoft Data Access Components (MDAC) 10.0, and the CLAS (Nutrition Institute, Ljubljana, Slovenia). Data processing was performed using Microsoft Excel 2019 (Microsoft, Redmond, Washington, DC, USA).

For statistical evaluation, we calculated sale-weighted and non-weighted proportions of foods fortified with VitD in different food categories, and the average VitD contents.

For the proportions of food fortified with VitD, we used a descriptive analysis and Wilson score interval (68). A 95% confidence interval (95% CI) is provided. A two-tailed *z*-test was used to identify increases in proportions in VitD fortification in 2020, compared to year 2017; negative value present lower proportion in 2017. Same statistical test was also used to compare within-category proportions of VitD fortified foods that pass WHOE nutrient profile in the 2017 dataset, in comparison with non-fortified foods. The level of significance was set at *p* < 0.05.

## Results and Discussion

Among 21,090 foods in the year 2017, VitD was added to 235 foods. In the 2020 dataset, VitD fortification was found in 270 out of 28,028 foods. All further results are reported for reduced datasets of food categories in which VitD fortification was observed (*N* = 13,393 and 16,064, for years 2017 and 2020, respectively).

**Figure 1:**
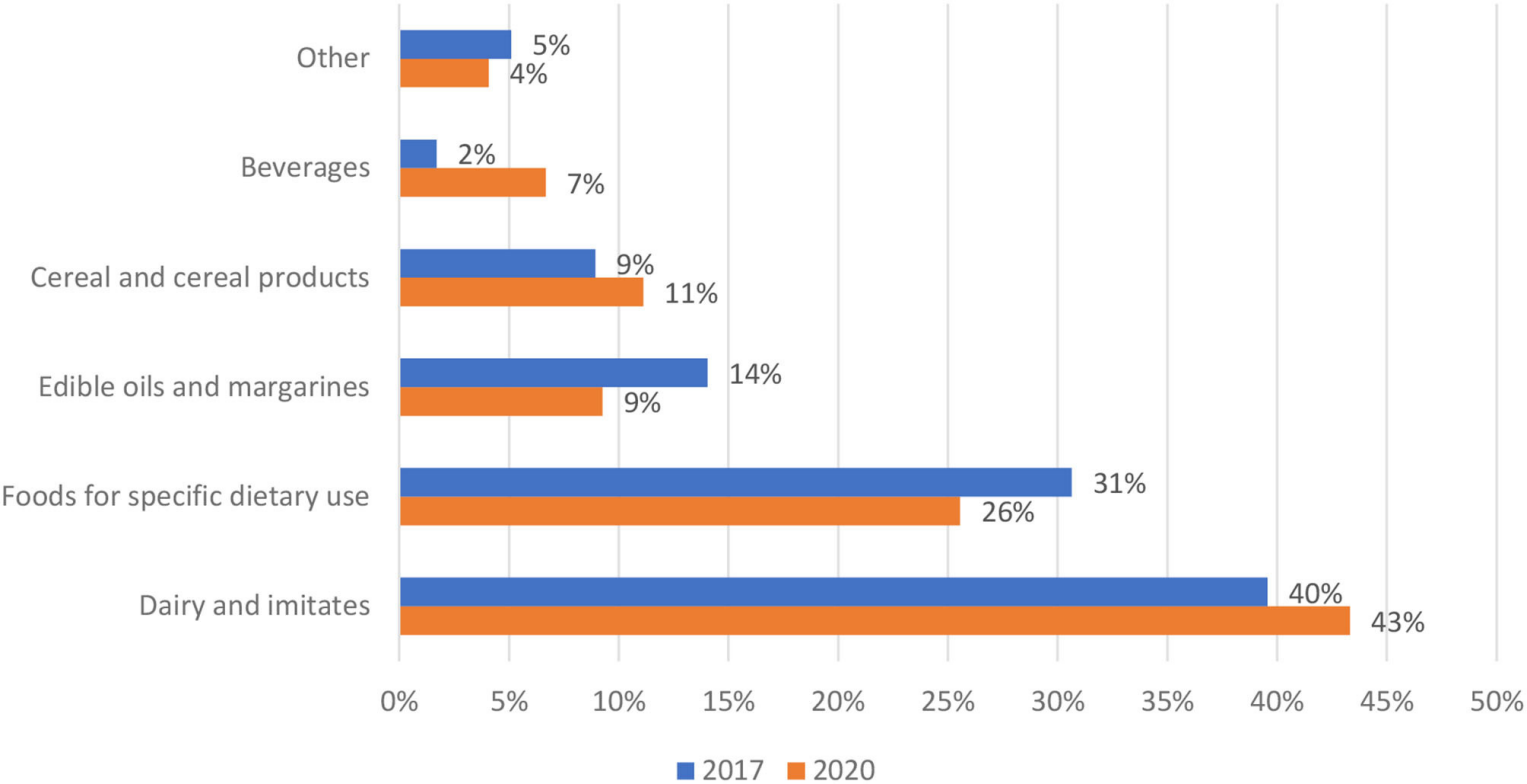
Distribution of prepacked foods fortified with vitamin D per food (sub)categories in years 2017 (*N* = 235) and 2020 (*N* = 270) (Slovenia).

The distribution of prepacked foods fortified with VitD in 2017 and 2020 is presented in Figure 1. In both years, the highest proportion of fortified foods was within dairy and imitates, which represent about two-fifths of the sample (40% in 2017; 42% in 2020), followed by foods for specific dietary use (for example, meal replacements, weaning foods, and infant formulas)-−31% in 2017 and 26% in 2020. In 2017, additional food subcategories with a notable proportion of fortification were edible oils and margarines (14%), cereal and cereal products (9%) and beverages (2%). In 2020, a somewhat higher proportion was observed in cereal and cereal products (11%) and beverages (7%), and lower in edible oils and margarines (9%). The remaining categories, each with less than a 3% share, are presented as combined (Figure 1, “other”); bread and bakery products (3.4% in 2017; 1.9% in 2020), sauces and spreads (0.9% in 2017; 1.1 in 2020) and confectionery (0.9% in 2017; 1.1 in 2020).

While each dataset presents cross-sectional data for a specific time period, a comparison of the same foods in both datasets provided very interesting insights into food reformulation practices. For this purpose, we matched products using the EAN barcode number as the product identifier. Focusing on the food categories in which VitD fortifications were observed, 6,534 foods were found in the datasets for both observation years, and 118 of those were fortified with VitD. In 109 (87.9%) of those products, we did not observe any changes in the VitD fortification practice in 2020, meaning that the content of added VitD did not change in these products in the observation period. However, in eight products (one from cereals, five from cakes, muffins, and pastry, and two from the chocolate and sweets category), the producers discontinued VitD fortification by 2020. We only found one product that did not have VitD fortification in 2017, but the manufacturer started adding VitD by 2020. These results do not indicate reformulation of foods by adding VitD to the existing formulations. Our data revealed that a higher number of VitD-fortified foods in 2020 corresponded with additional foods in the 2020 sample, that were not present in the 2017 dataset.

The penetration of VitD fortification in specific food (sub)categories is presented in Table 1. In both years, the largest share of VitD-fortified foods per category was represented by meal replacements (78% in 2017; 100% in 2020) and margarine, comprising about half of the sample (53% in 2017; 47% in 2020), followed by milk imitates (19% in 2017; 23% in 2020), yogurt imitates (21% in 2017; 19% in 2020), baby foods (18% in both years), flavored yogurt drinks (19% in 2017; 11% in 2020) and weaning foods (15% in 2017;11% in 2020). Except for breakfast cereals (5% in 2017; 6% in 2020), the share for other categories was <5%. It should be noted that the difference between both observation years was significant only in three food (sub)categories. Flavored yogurt drinks had lower proportions of VitD-fortified products in 2020, while the opposite was observed in beverages and meal replacements. In the latter case, beverages with added VitD were mostly soft drinks and energy drinks.

**Table 1:** (Sub)category proportions of foods fortified with vitamin D in the food supply for 2017 and 2020 (Slovenia).

**Food category**	**2017**					**2020**					**Two sample *z*-test for proportions**
	***N***	**VitD Cont. *N***	**% (95% CI)**	**Sale-weighted proportion (%)**	**Average VitD Cont. (μg per 100 g or ml)**	***N***	**VitD cont. *N***	**% (95% CI)**	**Sale-weighted proportion (%)**	**Average VitD cont. (μg per 100 g or ml)**	***p*-value**
Beverages	2,454	4	0.2 (0.0; 0.3)	0.03	1.6	3,385	18	0.5 (0.3; 0.8)	0.3	1.4	0.02
Bread and bakery products	2,105	8	0.4 (0.2; 0.8)	0.1	2.9	2,389	5	0.2 (0.0; 0.4)	/	^*^	ns
Cereal and cereal products	1,854	21	1.1 (0.7; 1.7)	1.0	4.3	2,196	30	1.4 (0.9; 1.9)	0.8	4.2	ns
– Breakfast cereals	375	18	4.8 (2.6; 7.0)	11.8	4.1	487	29	6.0 (3.9; 8.1)	9.9	4.3	ns
Confectionery	2,213	2	0.1 (-0.0; 0.2)	0.01	^*^	2,470	3	0.1 (0.0; 0.3)	/	4.2	ns
Dairy and imitates	2,843	93	3.3 (2.6; 4.0)	1.5	2.3	3,459	117	3.4 (2.8; 4.0)	2.2	1.9	ns
– Yogurt products	806	39	4.8 (3.4; 6.3)	4.4	1.1	911	39	4.3 (3.0; 5.6)	5.4	1.3	ns
– Flavored yogurt	419	10	2.4 (0.9; 3.8)	1.8	1.2	386	8	2.1 (0.7; 3.5)	4.5	1.3	ns
– Flavored y. drinks	119	22	18.5 (11.5; 25.5)	23.4	0.9	199	22	11.2 (6.7; 15.4)	21.0	1.0	0.03
– Plain yogurt	235	0	/	/	/	284	1	0.7 (−0.3; 1.6)	/	0.8	ns
– Yogurt imitates	33	7	21.2 (7.3; 35.2)	31.2	1.5	42	8	19.1 (7.2; 30.9)	33.3	1.1	ns
– Milk	114	4	3.5 (0.1; 6.9)	0.4	0.8	155	7	4.5 (1.3; 7.8)	0.3	0.9	ns
– Milk imitates	150	28	18.7 (12.4; 24.9)	10.6	1.0	185	42	22.7 (16.7; 28.7)	31.4	0.9	ns
Edible oils; oil emulsions	550	33	6.0 (4.2; 8.0)	8.6	7.1	617	26	4.2 (2.6; 5.8)	6.2	8.0	ns
– Margarines	62	33	53.2 (40.8; 65.7)	70.5	7.1	53	25	47.2 (33.7; 60.6)	65.8	8.1	ns
Foods for spec. dietary use	281	72	25.6 (16.6; 26.1)	9.8	6.1	251	69	27.5 (22.0; 33.0)	10.9	6.8	ns
– Baby foods	244	43	17.6 (12.8; 23.0)	8.8	6.7	222	40	18.0(13.0; 23.1)	10.5	7.7	ns
– Infant formula	15	15	100	100	9.3	20	20	100	100	13.3	ns
– Weaning foods	193	28	14.5 (9.5; 19.5)	4.4	6.4	180	20	11.1 (6.5; 15.7)	4.3	5.7	ns
– Meal replacements	37	29	78.4 (65.1; 91.6)	58.0	5.5	29	29	100	100	5.9	<0.01
Sauces and spreads	1,093	2	0.2 (-0.1; 0.4)	0.03	4.5	1,298	3	0.2 (0.0; 0.8)	0.5	1.5	ns

*VitD, Vitamin D; VitD cont. N, number of vitamin D-containing food products; Data presented for food categories with at least one product with VitD in either the 2017 or 2020 dataset. 95% CI-95% confidence interval; N, number of all products; ns, not significant; na, not applicable*;

* *data for VitD content was not available (VitD was declared only in the ingredients); / data not available*.

Considering that different prepacked foods have very different market shares, we calculate the per-category sale-weighted proportions of VitD fortification (Table 1). In this analyses we used 12-months sales data, provided by largest food retailers in Slovenia, covering the majority of the national market. It should be noted that the sales data were available for the majority of foods in both datasets (69% in 2017, and 68% in 2020), and for even more foods fortified with VitD (81% in 2017 and 82% in 2020). A general observation was that in most food categories with low proportions of VitD-fortified foods (<5%), the sale-weighted proportion of VitD fortification is even lower, while the opposite was observed in categories with higher proportions of fortification. The exceptions are foods for specific dietary use (where 25.6% were fortified in 2017 and 27.5% in 2020, corresponding to 9.8 and 10.9% of the market share, respectively), and milk imitates (in 2017, 18.7% of available milk imitates were fortified with VitD, but these only presented 10.6% of the market share). Interestingly, the situation changed notably in 2020, when the market share of VitD-fortified milk imitates increased to 31.4%. We should also mention some food (sub)categories, where sale-weighting resulted in very interesting observations. In both observation years, the market share of fortified breakfast cereals was almost double (11.8 and 9.9% in 2017 and 2020, respectively), in comparison with the proportion of such food on the market. Notable sale-weighing effects were also observed in fortified margarine (which presented 70.5 and 65.8% of the market share, respectively), yogurt imitates (31.3 and 33.3%, respectively), and flavored yogurt drinks (23.4 and 21.0%, respectively).

We also investigated the VitD content in fortified foods (Table 1). On average, more VitD was added in the categories of margarines and edible oils (7–8 μg/100g), foods for specific dietary use (6–7 μg/100g)—especially in infant formula (9–13 μg/100g), and breakfast cereals (4 μg/100g). It should be noted that some of these foods are typically consumed in smaller portions, <100 g per day. In foods that are typically consumed in portions above 100 g, lower levels of VitD were observed. For example, around 1 μg/100g in yogurts and imitates, and milk and imitates. The average VitD content in fortified beverages was 1.6 μg/100g in 2017, and 1.4 μg/100g in 2020. These results are quite comparable with the French study, which reported the VitD fortification of dairy products, breakfast cereals, edible oils, and margarines; the average VitD content varied from 0.8 μg/100g in dairy products to 8 μg/100g in margarines (69). And with a study covering several European countries, where the content of VitD in milk and dairy was 0.1–1.2 μg/100g, and 7–8 μg/100g in margarines (70).

**Figure 2:**
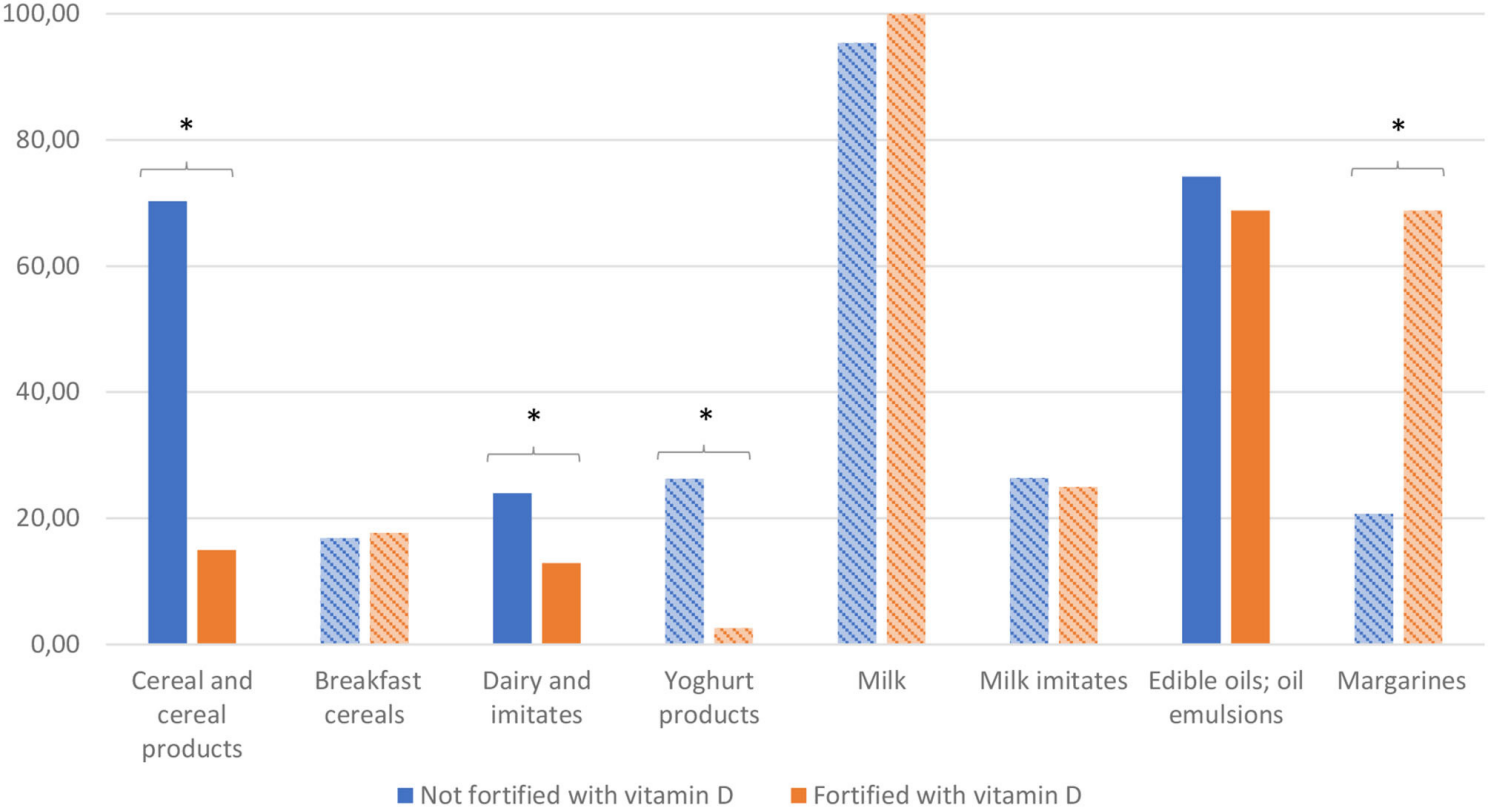
(Sub)category proportions (%) of (healthier) foods passing WHOE nutrient profile model for samples of foods (1) which are not fortified, or (2) are fortified with vitamin D. ^*^Significant differences. Subcategories are shown in bars using diagonal lines pattern. Profiling using the WHO Europe (WHOE) nutrient profile (62).

**Figure 3:**
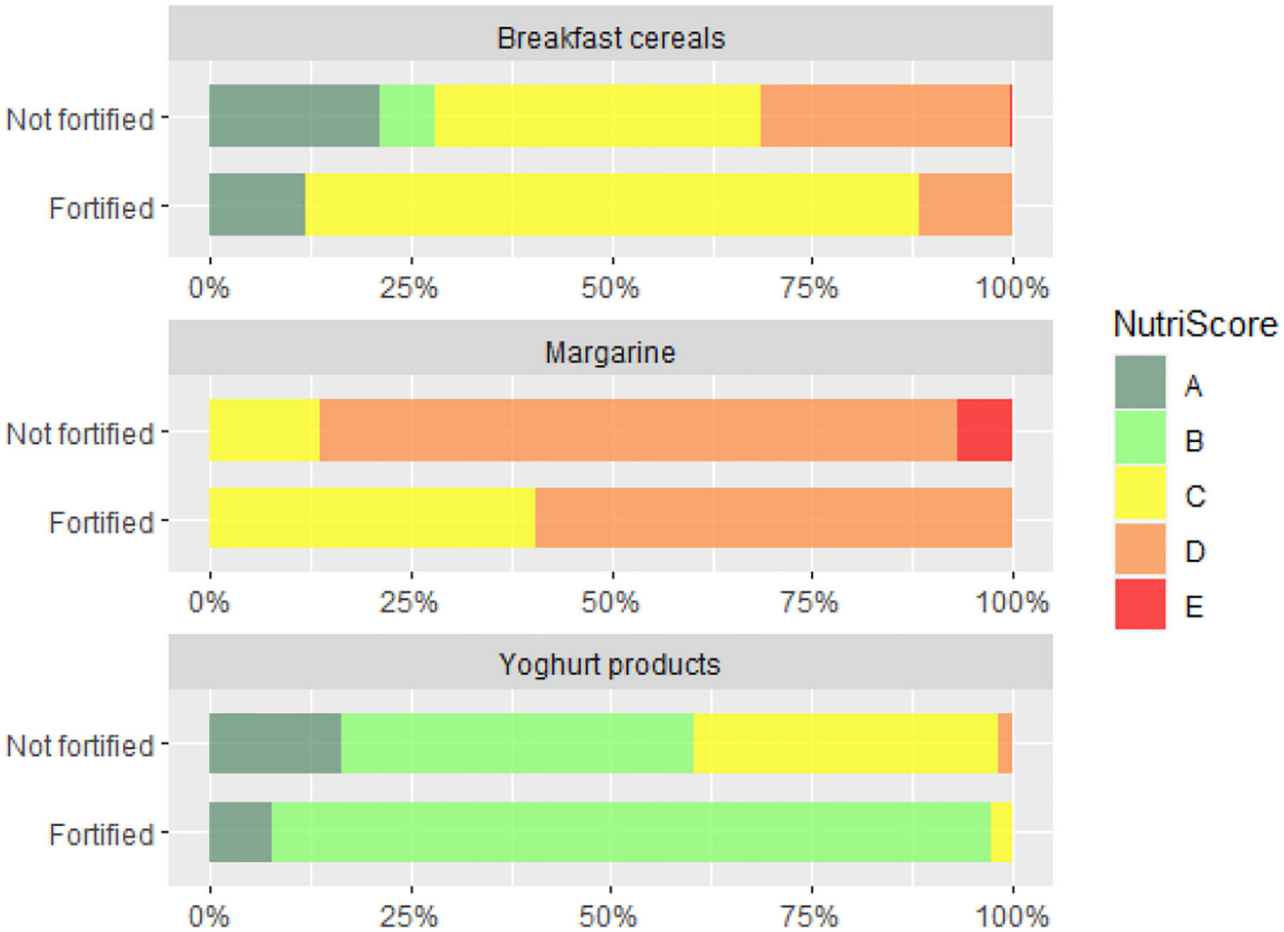
Distribution of Nutri-Score grades (A-B-C-D-E) in breakfast cereals, margarine and yogurt products which are (1) not fortified and (2) fortified with vitamin D.

Further we compared overall nutritional quality of the foods fortified with VitD with those, which were not fortified. Analyses was done using the 2017 dataset. First, we employed WHOE nutrient profiling model, which enable distinguishing foods of lower (less healthy) and higher (healthier) overall nutritional quality. Figure 2 provide results of analysis for the selected food (sub)categories, in which at least 2% of foods were fortified with VitD. To provide further insights, Nutri-Score (NS) nutrient profile model was also used, grading foods into five different grades of the overall nutritional quality (with dark green grade A appointed to those with highest nutritional quality, and grade E to those with lowest quality). Because such a comparison is relevant only in food subcategories with sufficient number of fortified products, Figure 3 presents comparison for breakfast cereals, margarine and yogurt products.

Profiling with WHOE showed significant lower nutritional quality of VitD fortified cereal and cereal products. When we take a closer look into a subcategory of breakfast cereals, which are typically ultra-processed foods, no significant differences were observed (Figure 2). However, the use of Nutri-Score revealed interesting observation, that vast majority of VitD fortified breakfast cereals is in the middle quality grade C, while in non-fortified foods both higher and lower grades are much more evenly distributed (Figure 3). Among dairy products and imitates, the WHOE also highlighted lower nutritional quality in subcategory of yogurts. This was due to very strict sugar content cut-off in the WHOE model for this category (10 g sugar/100 g food), which cannot be compensated with other parameters, i.e., lower (saturated) fat content. On the other hand, Nutri-Score—where final score is calculated with consideration of positive and negative nutrients, graded both fortified and non-fortified yogurts notably better. Interestingly, margarine was the only food category, where nutritional quality of fortified products was rated better than for non-fortified foods, by both WHOE and NS. However, promotion of excess consumption of such high fat foods not appropriate, which makes margarine a limited source of vitamin D for the general population. In other European countries, fat spreads, breakfast cereals, milk, and certain baby foods are most often fortified with VitD (71, 72). Our results also show that in both years, margarine was the most common food category fortified with VitD, and also contained the highest amount of this vitamin per 100 g. This observation is similar to the situation in the Netherlands, where margarine was also the most frequently consumed fortified food product (73). It should be noted that margarine with the highest market share in our study were from major European manufacturers, which are also sold in other EU markets. In Europe, the voluntary fortification of margarine has been practiced since 1925, but with the advent of the second world war, when margarine became a major replacement for butter (74), some European countries also required mandatory margarine fortification with VitD (75–77). In the UK, the mandatory fortification of margarine was revoked in 2014 (78), but most margarine are still voluntarily fortified.

Interestingly, while milk is also commonly fortified with VitD in some European countries (particularly in northern Europe), as well as in Canada and the USA—either voluntarily or mandatorily (79, 80)—this is not the case in Slovenia. Only about 4% of milk was fortified with this vitamin, and the market share of such products was much lower (<0.5%). This can be explained by the fact that in contrast to margarine, the majority of milk in Slovenia originates from local suppliers.

As in Europe and elsewhere in the world, challenges with VitD status have been observed among various population groups (81–88). The data from the 4-year (2013–2017) food-based solutions for optimal VitD nutrition and health through the life cycle (ODIN) project revealed that 1 in 8 (13%) of the EU population are VitD deficient, and 40% insufficient (85, 89). The situation can be much worse in seasons in which sun exposure is not sufficient for the biosynthesis of VitD in human skin. For example, during the winter in Slovenia, about 40% of the population was VitD deficient, while insufficiency was observed in 80% of the adult population (21). With the absence of VitD biosynthesis, sufficient dietary intake of VitD is needed, but typical intakes in most populations are rather low. For example, in Slovenia the estimated daily intake of VitD in adults is only 2.9 μg, much below the recommended intake of 20 μg/day (90–93). Also in Canada, the majority of the population consumed very low amounts of VitD, and mandatory fortification of milk was, therefore, implemented (94). However, except in one age group (1–50 years) VitD intake is still below the estimated average requirement (95), which is consistent with reports of low serum 25-OH-Vitamin D concentrations in different population groups (96–98). European food consumption surveys also show low VitD intakes in Europe (44, 99–103), with a very limited contribution of fortified foods (104). However, improvements in serum concentrations of 25-OH-Vitamin D and a lower prevalence of VitD deficiency were reported in a long-term study after 11 years of mandatory fortification in Finland (45). Similar observations were also reported in another Finnish study, 2 years after implementing the mandatory fortification of milk and margarine (42). The available data suggest that improving VitD status through food fortification (when implemented at the population level) is cost-effective (105).

All the findings point to the need to increase the intake of vitD through food in Slovenia, as it is difficult to expect people to change their diet in order to increase the intake of VitD with regular foods, rich in this vitamin, such as fish. On the other hand, increased consumption of other dietary sources of vitamin D (i.e., liver, fortified margarine, eggs) is also not in line with the dietary guidelines for healthy eating. While Slovenia has not implemented mandatory fortification of foods with VitD, a very recent Slovenian study has estimated changes in dietary VitD intake in a hypothetical scenario of mandatory milk fortification with 2 μg of VitD per 100 ml (93). Study results showed a notable increase in the predicted VitD intake, but the expected intake would be still much below the recommended intake. However, we should mention very positive experiences with food fortification in Slovenia. Mandatory iodisation of salt was implemented very successfully back in 1953 (106), addressing iodine deficiency (107). WHO also highlighted the importance of mandatory micronutrient fortification in the case of a high prevalence of deficiencies in certain populations (25).

Evaluation of the overall nutritional quality of the foods fortified with VitD showed differences between food categories; in many cases fortified foods had low overall nutritional quality, which can be explained with the fact that currently fortification is more commonly practiced among ultra-processed foods, which are typically having lower nutritional quality (108). Fortification enables food manufacturers to use nutrition and health claims, which can be very attractive to consumers and could mask overall poor nutritional quality of the food (109). According to the EU nutrition and health claims regulation, such practices should be prohibited with the introduction of nutrient profiles—back in year 2009 (110). While this part of the regulation has not yet been implemented (111), our study highlighted that this is very relevant. It should pointed out that our observations reflect the situation of fully voluntarily market-driven addition of VitD to foods. In case of mandatory fortification, VitD would be more commonly added to less processed foods with higher overall nutritional quality.

The strength of this study is in the use of the nationally representative cross-sectional branded food composition datasets at two time points (2017 and 2020), and in the use of sales data to address market share differences. Such an approach has been used in the past to investigate various public health risks [e.g., the content of salt (55), sugar (56), and additives (57)]. Some study limitations also need to be mentioned. While our datasets were representative of the national food supply market, the data collections were conducted in major retailers with shops distributed nationwide. This means that some foods that are only available in smaller local shops are not presented. However, such products have low market shares, and we do not expect that they would notably affect the study conclusions. We also did not have access to sales data for all products in our datasets, but it should be mentioned that the sales data were provided by major retailers, covering the majority of the national market. We should mention that majority of the VitD fortified foods were international brands, sold across the European Union, making study results very relevant for larger region. For example, less than 9% of the fortified foods in our dataset (*N* = 19 and *N* = 24, in the 2017 and 2020 dataset, respectively) were produced in Slovenia, while over 90% were imported. A limitation of our study is that the VitD content in foods was not determined in the laboratory, but taken from food labels. While we did not have the capacity for the analyses of the foods, further studies should also investigate this topic, particularly if mandatory fortification is introduced. We should note that previous studies highlighted that, due to the instability of VitD in certain food matrices, the amount of this vitamin in fortified foods can be lower than declared (112). We should also note that the addition of VitD to food does not necessarily mean that such product has a better nutritional composition, and nutrient profiling analyses was therefore applied. However, a limitation is, that nutrient profiling analyses was only done on the 2017 sample, because the 2020 dataset has not yet been updated with estimates for missing data on the content of certain nutrients/constituents, which are used in the nutrient profiling algorithms (i.e., dietary fiber content).

While previous research highlighted notable brand differences in the nutritional quality of foods (113–115), our dataset of VitD fortified foods was not large enough for such an analysis. However, this topic is very interesting for further research, which need to focus into larger datasets, for example into foods with added other vitamins and/or also minerals.

## Conclusions

We showed that branded food composition databases are extremely useful resources for the investigation and monitoring of food fortification practices, particularly if sales data can also be used. In Slovenia, the fortification of foods with VitD is fully voluntary, without any formal recommendations. The comparison of nationally representative branded food datasets compiled in cross-sectional studies in 2017 and 2020 showed minor differences between both years. Overall, the highest prevalence of fortification was observed in margarine, where VitD was added to about half of the products. Other food categories that are more commonly fortified with VitD are breakfast cereals, yogurts and their imitates, and foods for specific dietary use. The highest average content of VitD was observed in margarine. Major food-category differences were also observed in comparison of the overall nutritional quality of the fortified foods; higher overall nutritional quality was only observed in fortified margarine. In the absence of a mandatory or recommended fortification, very few manufacturers decide to add VitD, and even when this is the case, such products are commonly niche foods with lower market shares. We observed exceptions in imported foods, which can be subject to fortification policies introduced in other countries.

## Data Availability Statement

The raw data supporting the conclusions of this article will be made available by the authors, without undue reservation.

## Author Contributions

IP: conceptualization. MH and SK: data collection. IP and KŽ: methodology. EH: nutrient profiling. SK: formal analysis and writing—original draft preparation. MH, KŽ, and IP: manuscript review. All authors: manuscript writing—review and editing. All authors have read and approved the final version of the manuscript.

## Funding

The study was supported by the national research programme “Nutrition and Public Health” (P3-0395) and “Infrastructure programme for monitoring of the composition and labelling of foods” (IO-0054), funded by the Slovenian Research Agency; and the research project “Challenges in achieving adequate vitamin D status in the adult population” (L7-1849), funded by the Slovenian Research Agency and the Ministry of Health of the Republic of Slovenia. We also acknowledge support of the Nutrition Institute in the FNS-Cloud project, which received funding from the European Union's Horizon 2020 Research and Innovation programme (H2020-EU.3.2.2.3—A sustainable and competitive agri-food industry under Grant Agreement No. 863059).

## Author Disclaimer

The information and views in this report do not necessarily reflect the official opinion or position of the European Union. Neither the European Union Institutions and bodies nor any person acting on their behalf may be held responsible for the use of the information contained herein.

## Conflict of Interest

We acknowledge that IP has led and participated in various other research projects in the areas of nutrition, public health, and food technology, which were (co)funded by the Slovenian Research Agency, Ministry of Health of the Republic of Slovenia, the Ministry of Agriculture, Forestry and Food of the Republic of Slovenia and, in cases of specific applied research projects, also by food businesses. IP and KŽ are members of a National Workgroup responsible for the development of recommendations for assuring adequate vitamin D status among the Slovenian population. The authors declare that this research was conducted in the absence of any commercial or financial relationships that could be construed as potential conflicts of interest.

## Publisher's Note

All claims expressed in this article are solely those of the authors and do not necessarily represent those of their affiliated organizations, or those of the publisher, the editors and the reviewers. Any product that may be evaluated in this article, or claim that may be made by its manufacturer, is not guaranteed or endorsed by the publisher.
